# Weed Out the Many Chemistries of Cutin Oligomeric Mixtures: Developing Antimicrobial Films by Blending with Carboxymethylcellulose

**DOI:** 10.1021/acssuschemeng.6c01134

**Published:** 2026-04-15

**Authors:** Rita Escórcio, Artur Bento, André Cairrão, Enkeledo Menalla, Erika Zamboni, Constanza Maciel, Mathieu Fanuel, Bénédicte Bakan, Annamaria Celli, María José Cocero, Cristina Silva Pereira

**Affiliations:** † Instituto de Tecnologia Química e Biológica António Xavier, Universidade Nova de Lisboa (ITQB NOVA), Av. da República, 2780-157 Oeiras, Portugal; ‡ Department of Chemical Engineering and Environmental Technology, BioEcoUva, Research Institute on Bioeconomy, 16782 University of Valladolid, Calle Doctor Mergelina S/N, Valladolid 47011, Spain; § Department of Civil, Chemical, Environmental and Materials Engineering, 9296 University of Bologna, Via Umberto Terracini, 28, 40131 Bologna, Italy; ∥ 27057 INRAE, UR 1268, Biopolymers, Interactions & Assemblages (BIA), F-44316 Nantes, France; ⊥ PROBE Research Infrastructure, BIBS Facility, INRAE, F-44316 Nantes, France

**Keywords:** tomato pomace, ionic liquids, supercritical water (SCW), biobased materials, cutin, bioeconomy

## Abstract

Valorization of agro-industrial residues like tomato pomace is supported by the growth of a circular economy. Tomato pomace is a widely used source of the cutin polyester, a raw material for creating biobased materials and coatings. Cutin is usually incorporated into materials in the form of its hydrolyzable products. In this study, we focused on the production of biopolymer films composed of carboxymethylcellulose (CMC) and cutin oligomeric mixtures (COMs) yielded by chemical or supercritical water hydrolysis (SCWH). Either COM and their derived materials were comprehensively characterized (e.g., NMR, GC–MS, MALDI-TOF MS), including antimicrobial properties against *E. coli* and *S. aureus*, two foodborne pathogens. The results show that despite their compositional differences, both COMs reported similar behavior in direct antimicrobial activity: kill ≤92% of *S. aureus cells* but promote *E. coli* growth. The materials comprising COMs showed variable hydrophobicity and reduced water uptake compared to controls. The COMs improved the materials killing efficacy against *S. aureus*, regardless of the fact that only those yielded by SCWH (richer in monomers) showed a concentration-dependent effect. All tested materials failed to inhibit the adhesion of *S. aureus*, and while COMs addition did not improve the materials’ capacity to kill *E. coli*, it effectively blocked its adhesion.

## Graphical abstract

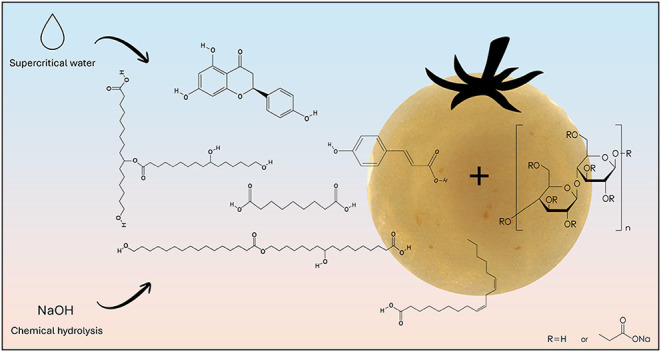

## Introduction

The handling and processing of agro-industrial residues poses major challenges due to their high abundance and negative environmental impacts.^1^ These residues are commonly discarded through incineration or landfilling, processes that generate significant greenhouse gas emissions.^2^ In recent years, the growth of circular economy has enabled these residues to re-enter production chains instead of being discarded. One example is tomato pomace, an agro-industrial residue from the tomato processing industry, composed of peels, seeds, and stems of tomatoes.^3–6^ This residue has already been used to produce animal feed, compost, and bioenergy, reaching technology readiness levels (TRL) between 7 and 9.^1^ Despite these applications, tomato pomace has the potential for further valorization processes if cascading biorefinery principles are implemented, given its richness in a wide variety of biomolecules (e.g., lipids, proteins, polysaccharides).^1,2,4,7^ This rationale is the backbone that supports the creation of biorefineries and enables the circular bioeconomy to reach its full potential.^1,2^

Tomato peel constitutes a rich source of cutin,^4,8,9^ a branched plant polyester composed of C_16_ and/or C_18_ ω-hydroxy acids that are linked through primary and secondary ester bonds.^10,11^ The heterogeneous composition of tomato pomace, containing the polymer cutin intermixed with various components, has historically hindered its use as a feedstock for cutin isolation. We previously demonstrated that the ionic liquid cholinium hexanoate can selectively isolate the polymer cutin from tomato pomace, overcoming, in a single-step, the inherent complexity and heterogeneity of the feedstock, hence supporting cutin downstream applications.^12–14^

As recently reviewed by Escórcio et al., cutin (largely obtained from tomato pomace and tomato peels) is typically hydrolyzed with an alkaline base (e.g., sodium hydroxide) to release its composing hydrolyzable constituents (mostly monomers).^15^ This enhances processability, for example, in the production of biobased materials as it allows a more homogeneous mixing with other polymer matrixes (e.g., chitosan and pectin^16,17^ ). In contrast to harsh hydrolysis of tomato cutin, which yields mostly monomers, we previously demonstrated that mild hydrolysis produces cutin oligomeric mixtures (COMs) where several monomer units remain linked, preserving some of the structural properties of cutin.^12^ These COMs exhibit promising antimicrobial activity, either broad (active against both Gram-positive and Gram-negative bacteria) or narrow (targeting only Gram-positive bacteria) with apparently distinct modes of action due to compositional differences.^12^ Moreover, some COMs elicited plant immunity.^18^ Recently, Enkeledo et al. demonstrated that supercritical water hydrolysis (SCWH) of tomato cutin yields hydrolysates also comprising oligomers.^19^ This methodology has the advantage of upscaling COMs production to a pilot scale, which is critical to advance the TRL of cutin usage.

Carboxymethylcellulose (CMC) is a water-soluble cellulose synthesized through reaction with alkali and chloroacetic acid.^20^ CMC is useful for thickening, suspending, and stabilizing solutions and can form tough films when the solutions are evaporated.^21^ Various studies have reported its use as a polymer matrix for antimicrobial applications in nanomaterials, hydrogels, and films.^22–25^ CMC is particularly suitable as a polymer matrix for blending with COMs since both contain free hydroxyl and carboxyl acid groups that can be linked through esterification reactions (*e.g*. polycondensation). Biodegradable films comprising CMC and extracts/powder of apple peels have been reported before focusing on their capacity to release antimicrobial and antioxidant additives rather than exploring cutin as a source of antimicrobial building blocks.^22^ Polycondensation has been explored to produced materials composed of cutin hydrolysates (mostly its main monomer) and glycerol (cross-linking agent), but these were unable to kill bacteria, even though addition of glycerol potentially reduced bacterial adhesion, particularly of *S. aureus*.^26^ Most previous studies where cutin hydrolysates were used for producing materials did not detail their composition nor their potential antimicrobial properties.^15^ One exception focused on chitosan films containing cutin hydrolysates, but only chitosan was considered antimicrobial.^27^ Our study aims to start solving these outstanding questions: can cutin hydrolysates be explored to produce antimicrobial materials, and can we identify basic design rules to potentiate antimicrobial activity?

In this study, biopolymer films composed of the CMC and COMs were developed. A homogeneous cutin raw material was obtained from tomato peels using an ionic liquid extraction process. COMs with different compositional signatures were produced by two different methodologies: chemical hydrolysis with sodium hydroxide and SCWH. The materials were prepared using three different concentrations of COMs from each methodology (10, 20, and 30% w/w) with pure CMC as the control. The COMs and the resulting materials were chemically characterized, mostly by spectroscopy and spectrometry techniques. The antimicrobial and antibiofouling capacity of the materials was evaluated against two representative foodborne bacteria: *Escherichia coli* and *Staphylococcus aureus*. Additional properties such as water swelling (affecting barrier performance), surface hydrophobicity (influencing bacterial adhesion), and thermal properties (determining processing windows and application stability) were also analyzed. The potential contribution of this study to the development of sustainable biodegradable alternatives to synthetic antimicrobial films is discussed in detail.

## Materials and Methods

### Chemicals

Sodium hydroxide (>98%) was obtained from José Manuel Gomes dos Santos; methanol (≥99.8%), ethanol (≥99.8%), acetone (≥99.8%), dichloromethane (>99.99%), sodium sulfate anhydrous (99%) were obtained from Fisher Chemical; cholinium hydrogen carbonate (∼80% in water), hexanoic acid (>99.5%), hydrochloric acid (37%), carboxymethylcellulose sodium salt were obtained from Sigma-Aldrich. Cholinium hexanoate was synthesized by dropwise addition of hexanoic acid to aqueous cholinium hydrogen carbonate in equimolar quantities, as previously described.^28^

### Plant Material

Tomato peels were obtained from TOMAPAINT (Parma, Italy). The dried peels were milled using a Retsch ZM200 electric grinder (granulometry 0.5 mm; 10,000 rpm) and stored at room temperature.

### Preparation of Cutin-Rich Materials

Cutin polymer was extracted from tomato peels as previously described.^12,13^ The peels and cholinium hexanoate were mixed (1:10 w/w) and stirred for 2 h at 100 °C. The reaction was stopped by the addition of excess ethanol (80 mL per g of peels). The polymer was recovered by filtration using a nylon membrane (0.45 μm) and washed with an excess of deionized water. The ethanol was recovered from the filtrate by distillation in a Rotavapor R-100 apparatus (BUCHI). The cutin samples were dried in an oven at 40 °C and stored at room temperature.

### Sodium Hydroxide Hydrolysis

The hydrolysis of the ionic liquid extracted cutin occurred by mixing 0.5 g of cutin with 20 mL of 1 M NaOH in methanol/water (1:1, v/v) at 90 °C for 1 h without stirring. The methanol/water mixture enables better dispersion of cutin and solubilization of its hydrolysates while facilitating ester hydrolysis, even when sterically hindered by polymeric organization.^29,30^ Each mixture was cooled to room temperature and centrifuged (4 °C, 30 min, 4000*g*) to remove the nonhydrolyzed cutin fraction (pellet). The supernatant was acidified to pH 3–3.5 with HCl 37% and centrifuged (4 °C, 30 min, 4000*g*). The precipitate (COM^1P^) was recovered, dried under a nitrogen flux, and stored at 4 °C, for further analysis.

### Supercritical Water Hydrolysis

Cutin slurry was prepared in a 2 L polypropylene tank (T-201) at a 5% (w/v) concentration in water, as previously described.^19^ A Milton Roy MD140 membrane pump (P-201) delivered water at 4 kg·h^–1^, while a Lewa EK1 piston pump (P-101) fed the cutin at 1.5 kg·h^–1^. Both streams were pressurized to 25 MPa and mixed, allowing hot, pressurized water to rapidly heat the slurry and initiate depolymerization. The reaction time (9.2 ± 0.3 s) was determined by the reactor length (175 cm) and the combined flow rates. The operating temperature (380 ± 5 °C) and pressure (250 ± 7 bar) were adjusted as required during operation. At the end of the reaction, the mixture was centrifugated in a Magnus 22R centrifuge from Ortoalresa (20 °C, 30 min, 3122*g*), and the precipitated (COM^PW^) was recovered, dried under a nitrogen flux, and stored at 4 °C, for further analysis.

### NMR

NMR spectra of COM^1P^ and COM^PW^ were recorded by using an Avance III 800 MHz CRYO (Bruker Biospin, Rheinstetten, Germany). All NMR spectra (^1^H, ^1^H–^13^C HSQC) were acquired in DMSO-*d*_6_ using 5 mm diameter NMR tubes, at 25 °C as follows: 15 mg of each sample in 400 μL of DMSO-*d*_6_. MestReNova, Version 11.04–18998 (Mestrelab Research, S.L.) was used to process the acquired raw data. All samples were analyzed in biological triplicates.

### GC-MS

To quantify the amount of free and hydrolyzable monomers present in each COM, an Agilent GC (7820A) equipped with an Agilent (5977B) MS (quadrupole) was used. Samples were derivatized (see below) directly or after alkaline hydrolysis (0.5 M NaOH in methanol/water (1:1, v/v), 95 °C, for 4 h; cooled to room temperature and acidified to pH 3/3.5 with 1 M HCl (37%), then extracted three times with dichloromethane/water partition). For derivatization, N,O-bis­(trimethylsilyl)­trifluoroacetamide containing 1% of trimethylchlorosilane in pyridine (5:1) was used (30 min, 90 °C). The ensuing samples were analyzed by GC–MS (HP-5MS column) with the following ramp temperature: 80 °C, 2 °C/min until 310 °C for 15 min. MS scan mode, with a source at 230 °C and electron impact ionization (EI+, 70 eV), was used for all samples. The GC–MS was first calibrated with pure reference compounds (heptadecanoic acid, hexadecanedioic acid, and benzoic acid) relative to hexadecane (internal standard). Triplicates, each with technical duplicates, were analyzed (6 samples per condition). Data acquisition was accomplished by MSD ChemStation (Agilent Technologies); compounds were identified based on the equipment spectral library (Wiley-NIST) and references relying on diagnostic ions distinctive of each derivative and its spectrum profile.

### MALDI-TOF Analyses

The samples were analyzed by laser desorption/ionization (LDI)-time-of-flight (TOF) MS and by matrix-assisted laser desorption/ionization (MALDI)-time-of-flight (TOF) MS. For the MALDI-TOF analyses, the analyses conditions were identical to those described by Moreira et al., as they were effective for observing both cutin monomers and oligomers.^18^ Samples were mixed with the matrix solution composed of DHB (2,5-dihydroxybenzoic acid) 3 mg·mL^–1^ in 75% methanol, with 2.5 mM LiCl, in a 1:3 ratio (v/v). The mixture (1 μL) was deposited on a polished steel MALDI target plate. Measurements were performed on a rapifleX MALDI-TOF spectrometer (Bruker Daltonics, Bremen, Germany) equipped with a Smartbeam 3D laser (355 nm, 10,000 Hz) and controlled using the Flex Control 4.0 software package. The mass spectrometer was operated in the reflectron mode with positive polarity. Spectra were acquired in the range 180–5000 *m*/*z*.

### HPSEC (High-Performance Size-Exclusion Chromatography)

The analysis utilized two styrene–divinylbenzene copolymer gel columns with pore sizes of 1000 and 500 Å, a mobile phase flow rate of 0.8 mL·min^–1^ of tetrahydrofuran (THF), and consistent column and detector temperatures of 40 °C (Aglient 1260 Infinity ii coupled with an IR detector and a DAD-UV detector). An injection volume of 25 μL was used. For sample preparation, 100 mg of sample was dissolved in 10 mL of THF, homogenized for 15 min, and filtered through a 0.22 μm nylon filter. Calibration standards included polystrene (reference number PL2010–0501), which served as a reference for determining the total molecular weight of the oligomeric mixtures.

### Cellulosic Materials Blended with COMs

Materials containing a mixture of 10, 20, and 30% (w/w) of COM^1P^ or COM^PW^ in CMC were prepared. Cutin oligomeric mixtures were first homogenized in acetone, CMC was dissolved in bidistilled water, and then a solution of 0.01 M HCl was added until pH 3 was reached. The two solutions were mixed and transferred to Teflon molds (40.7 cm^3^) and placed overnight at room temperature (20–25 °C), then heated in an oven at 40 °C for 16 h followed by another heating of 16 h at 150 °C. The materials were washed with 70% ethanol to remove nonbonded components, dried at 40 °C overnight, and stored at room temperature. Images of the materials were taken in a Zeiss stemi DV4 with an amplification of 13x.

### Attenuated Total Reflectance–Fourier Transform Infrared Spectroscopy (ATR-FTIR)

Infrared spectra of all of the materials were obtained with an Attenuated Total Reflectance (ATR) coupled to a Fourier Transform Infrared (FTIR) spectrometer accessory Invenio R Bruker. Spectra were recorded in transmission mode in the range 4000–600 cm^–1^ with 4 cm^–1^ resolution and accumulating 128 scans analyzed by OPUS software (version 8.7.41). Triplicate measurements were made, and a representative spectrum was chosen.

### Absorptive Wetting/Swelling Test

The method was adapted from a previous study.^8^ The initial weights of the materials with dimensions of 0.5 × 2 cm (1 cm^2^) were noted. The samples were fully immersed in 6 mL of bidistilled water; and after 120 min, removed, then patted dry, and finally weighted again. The test was performed in triplicates at room temperature. The following formula was used to calculate the swelling percentage

1 $$\%\;\mathrm{swelling}=\frac{f\mathrm{ilm weight after swelling}-i\mathrm{nitial weight of the film}}{i\mathrm{nitial weight of the film}}\times 100$$

### Water Contact Angle (WCA)

Contact angles were measured to determine the hydrophobicity of the materials using a drop shape analyzer DSA30 KRUSS (Germany). Measurements were conducted at room temperature by depositing 4 μL droplets of distilled water onto the materials. At least five measurements were collected for each sample (1 independent film tested; 9.89 cm^2^). The measurements were made as soon as the droplet landed on the material (0–1 s).

### DSC

The DSC analyses of the materials were done in a differential scanning calorimeter DSC4000 (PerkinElmer). Before performing the measurements, the materials were dried at 60 °C overnight under a vacuum. A temperature interval of −70 to 200 °C was studied, performing a first heating scan at 20 °C·min^–1^, 1 min of isotherm at 200 °C, and a cooling scan and a second heating scan at 10 °C·min^–1^. The samples weighed around 4 mg and were sealed in aluminum pans before the experiment.

### Antimicrobial and Antibiofouling Assays

*S. aureus* NCTC8325 and *E. coli* TOP 10 cells (5 × 10^5^ cells·mL^–1^) in Mueller–Hinton broth (MHB) media were exposed for 24 h to 7.9 mm^2^ of materials or to 1000 μg/mL of COMs at 37 °C under orbital agitation (100 rpm). After 24 h, the antimicrobial effect was evaluated through colony-forming units (CFU). To test the washed CMC materials, films containing a 7.9 mm^2^ area were transferred to a plate insert (6.5 mm with 0.4 μm membrane pore) and the conditions were set as described before against *S. aureus* NCTC832 cells. All assays were done with three biological replicates having a distinct bacterium inoculum (each with technical replicates: 9 > *n* ≥ 3).

### SEM

The materials exposed to the bacterial cells for 24 h (same conditions as above) were collected to scan the presence of adherent bacterial cells on the surface. The films were first washed with phosphate-buffered saline to remove nonadherent bacteria, subsequently fixed with glutaraldehyde 2.5% (v/v) during 10 min. After 10 min, samples were rinsed with distilled water and dehydrated with aqueous solutions with increasing concentrations of ethanol (ranging from 70 to 100%). Samples (including films prior to bacterial exposure) were placed on an aluminum stub using double-sided carbon tape and sputter coated with a thin Au film in a Quorum Technologies coater, model Q150T ES. Scanning electron microscopy (SEM), the microscope JEOL JSM-7001 F with an accelerating voltage set to 15 kV, or the microscope Phenom ProX G6 (ThermoScientific) were used to scan the film’s surfaces.

### Statistical Analyses

All of the data sets regarding the analysis of WCA, swelling, and the antimicrobial activity of the materials passed through an identical pipeline: a Shapiro-Wilk normality test was first performed to assess if the data had a normal distribution, followed by a Levene’s test to determine the homogeneity of variances. The data sets with a normal distribution were analyzed by a one-way analysis of variance (ANOVA) coupled with Dunnett’s Test for pairwise comparison with the CMC blank (*p* < 0.05*; <0.01**; <0.001***; <0.0001 ****). The data sets without a normal distribution were analyzed by Kruskal–Wallis test coupled with Dunn’s posthoc for pairwise comparison with the CMC blank (*p* < 0.05*; <0.01**; <0.001***; <0.0001****). The statistical analysis of the GC-MS data evaluated the differences in the monomeric content between COM^1P^ and COM^PW^ samples. A Welch’s two-sample *t* test was performed for each of the 12 monomers, using the mean values and the standard deviations. All of the statistical analyses and the heatmap generation were performed using the R studio version 2025.09.1 + 401 (R version 4.5.0).

## Results and Discussion

### Isolating the Raw Material

Tomato peels obtained by a flotation process of the tomato pomace were subjected to the cholinium hexanoate isolation as described previously.^12–14^ Aiming to increase the sustainability of the process, DMSO (used to stop the reaction and reduce viscosity) was replaced by ethanol. This optimization reduced the time required to filter cutin to ∼3 h and allowed the recovery and reusability of both ethanol (by distillation; recovery rate ranging between 80 and 85%) and the ionic liquid for another round of cutin isolation (obtaining a similar isolation yield: ∼80%). The isolated cutin polymer showed a similar NMR profile of those isolated by the same methodology from different tomato residues, having the archetypal signals of an aliphatic backbone (CH_2_) composed of ester bonds (primary aliphatic esters- PAE and secondary aliphatic esters- SAE), hydroxyl (−OH) and carboxyl groups (−COOH) (Figure S1).

### Chemical Characterization of the COMs

The ensuing ionic liquid isolated cutin was used to produce COMs by using either NaOH (COM^1P^) or SCWH (COM^PW^). The ^1^H–^13^C HSQC spectra of COM^1P^ and COM^PW^ show the presence of oligomeric features with the assignment of PAE and SAE (Figure 1). Methyl-esters (ME: CH_3_-ester) are only present in COM^1P^ and are a byproduct of the hydrolysis that contains methanol in the aqueous solution. Other assignments from free acid groups (α (C=O) acids) and free hydroxyl groups, both midchain and terminal (CH_2_CH-(OH); CH_2_CH_2_OH) are present in both samples. Regarding the relative quantification of the ester configurations (by ^1^H NMR) both COMs had similar values between SAE (52 to 59%) and PAE (35 to 41%), adding the MEs (12%) in COM^1P^ as previously discussed (Table S1). This relative quantification was performed by the addition of an internal standard (benzene) to the samples and integration of the respective peak areas (SAE/PAE) in the ^1^H spectrum.

**Figure 1:**
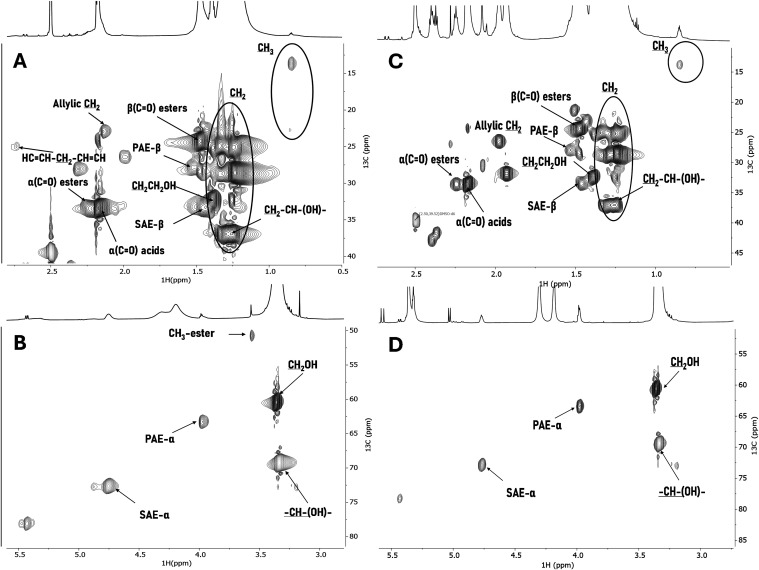
NMR spectral characterization of cutin oligomeric mixtures. ^13^C–^1^H HSQC spectral characterization of COM^1P^ (A, B) and COM^PW^ (C, D) detailing the aliphatic (A, C) and glycerol CH-acyl (B, D) regions. Some assignments (unlabeled) are uncertain or unidentified.

Despite the oligomeric features, COM^1P^ and COM^PW^ contained free monomers *c.a*. 14.8 and 30.5 wt %, respectively (GC–MS analyses, Table S2). The proportion of free monomers (%) was calculated according to the formula: (Non-Hydrolyzed Monomers/Total Monomers) × 100, where the total monomers comprise both free and ester-linked monomers within the oligomers. The GC-MS analysis of both samples, after a full depolymerization, showed a distinct profile with only 3 monomers being in similar quantities between the samples: 4-coumaric acid, naringenin, and nonanedioic acid (Table 1, marked with *). The most abundant family of compounds was ω-hydroxyalkanoic acids, and the most abundant monomer within was 10,16-dihydroxyhexadecanoic acid (485.4 mg/g (∼86.2% of the total monomers identified) and 766.6 mg/g (∼87.2% of the total monomers identified) for COM^1P^ and COM^PW^, respectively). These results are in accordance with the literature since this family of compounds can represent between 16 to 92% and 10,16-dihydroxyhexadecanoic acid between 64 to 82% of the composition from tomato cutin.^31^

**Table 1:** Quantitative Analysis of the Total Hydrolysable Monomers of the COMs by GC-MS^a^

compounds	COM^1P^ (mg/g)	COM^PW^ (mg/g)
**alka(e)noic acids**	**16.34 ± 1.4**	**36.71 ± 16.59**
hexadecanoic acid	5.32 ± 0.9	15.73 ± 0.4
9,12-octadecadienoic acid	3.86 ± 0.23	13.5 ± 0.07
9-octadecenoic acid	3.65 ± 0.14	13.24 ± 0.02
octadecanoic acid	3.52 ± 0.14	14.49 ± 0.16
**ω-hydroxyalkanoic acids**	**485.38 ± 62.43**	**766.59 ± 80.12**
16-hydroxyhexadecanoic acid	13.35 ± 4.16	42.44 ± 3.17
10,16-dihydroxyhexadecanoic acid	452 ± 58.29	724.15 ± 77.17
9,10-epoxy-18-hydroxyoctadecanoic acid	20.03 ± 4.18	0 ± 0
**α, ω-alkanedioic acids**	**1.66 ± 0.64**	**13.47 ± 15.09**
nonanedioic acid	0.17 ± 0.12*	13.22 ± 15.77*
hexadecanedioic acid	1.49 ± 0.52	27.98 ± 0
**aromatics**	**21.12 ± 5.56**	**22.58 ± 17.23**
4-hydroxybenzaldehyde	1.47 ± 0.41	17.25 ± 4.18
4-coumaric acid	17.49 ± 5.69*	12.75 ± 6.33*
naringenin	2.16 ± 1.73*	0.46 ± 0.12*
**identification yield (%)—area**	**84.91 ± 3.91**	**65.33 ± 1.03**

a Results are given as milligrams of compound per gram of starting material. The identification yields are indicated below and represent the ratio between the identified peak area and the total area of the peaks in the chromatogram. Monomers that were not detected in a specific sample are labeled as n.d.. Monomers having no statistical difference between the samples are marked with an asterisk (Welch’s two-sample t-test).

The MALDI-TOF analysis identified 11 different oligomers in COM^1P^, ranging from dimers to tetramers, illustrating the structural diversity generated by mild cutin hydrolysis (Table S3). Although the data are qualitative, the signal-to-noise ratio (S/N) reported in Table S3 provides insights into the most abundant species, with three dimers representing the top three oligomers: a dimer of 10,16-dihydroxyhexadecanoic acid, a dimer of hydroxyhexadecanedioic acid linked to naringenin, and a dimer of hexadecanedioic acid linked to naringenin (likely all via ester bonds).

The ratio of monomers after and before hydrolysis in the GC-MS (H/NH ratio) indicates that 10,16-dihydroxyhexadecanoic acid is one of the major building blocks of oligomers (Figure S2), with hexadecanedioic acid and naringenin also present in many configurations. The MALDI-TOF analysis for COM^PW^ identified seven different oligomers, with three dimers being the most abundant species: a dimer of 10,16-dihydroxyhexadecanoic acid, a dimer of 10,16-dihydroxyhexadecanoic acid linked to hexadecanedioic acid, and a dimer of hexadecanedioic acid linked to 16-hydroxyhexadecanoic acid (Table S3). The H/NH ratio of monomers further supports the MALDI-TOF results while additionally pointing to 4-coumaric acid and 4-hydroxybenzaldehyde as contributing building blocks of the oligomers (Figure S2). The presence of the monomer 4-hydroxybenzaldehyde is not usual for tomato cutin and can reflect an artifact or a degradation product from the oxidative degradation of phenolic precursors, such as *p*-coumaric acid or other aromatic compounds.^32^ 4-Hydroxybenzaldehyde is ∼10-fold higher in the oligomeric mixture produced using SCWH compared to that of the standard hydrolysis, likely due to the critical conditions used (e.g., operation temperature 380 ± 5 °C).^33^ We have detected minor amounts of this compound in a previous study,^12^ therefore one cannot disregard that it can be formed during sample processing and analysis.

The HPSEC profiles of TFH-soluble fractions of COM^1P^ and COM^PW^ were very similar, with both samples displaying a number-average molecular weight (Mn) of approximately 0.4 kDa and a weight-average molecular weight (Mw) of 0.7 kDa, and polydispersity indexes (PDI) of 1.83 and 1.76, respectively (Figure S3). This similarity indicates that the oligomeric species present in the THF-soluble fractions are comparable in size distribution. However, COM^1P^ and COM^PW^ differ in their insoluble components: COM^PW^ was fully soluble in THF, while COM^1P^ was only partially soluble.

Overall, the results show that the NaOH methodology produces higher amounts and a greater diversity of oligomers than SCWH, as evidenced by GC-MS, MALDI-TOF, and PDI results. SCWH of cutin yielded a higher proportion of free monomers, with the most abundant being 10,16-dihydroxyhexadecanoic acid, which can, in principle, form linkages through both its hydroxyl and carboxyl groups. For example, the carboxyl groups can react under standard polycondensation conditions with the hydroxyl groups of CMC and also with other cutin monomers and oligomers. Finally, all oligomeric species detected have free end groups that are prone to undergo polycondensation as well.

### Blends of CMC and COMs

The COMs were blended with CMC in three different concentrations: 10%, 20%, and 30% w/w (Figure 2). The preparation of the materials follows a 16 h polycondensation process at 150 °C to promote the polymerization between CMC and the COMs, while evaporating the water formed as a subproduct. The reactions promoted at lower temperatures (80 and 100 °C) resulted in brittle films (data not shown). In addition, several rounds of optimization were tested, mostly to avoid the formation of irregularities at the materials’ surface. This was achieved by introducing a temperature gradient (25 °C = >40 °C = >150 °C) to slow the evaporation of the acetone. Finally, an ethanol washing step was added to remove most of the unbound constituents (Table S4). The average percentage of mass loss per CMC film was 4%. We noticed that in the materials containing CMC and COM^1P^ the mass loss decreased proportionally with the increase of the COM concentration (from 10.9 to 5.7%), suggesting a more efficient esterification process. This optimized process was then applied to the remaining materials.

**Figure 2:**
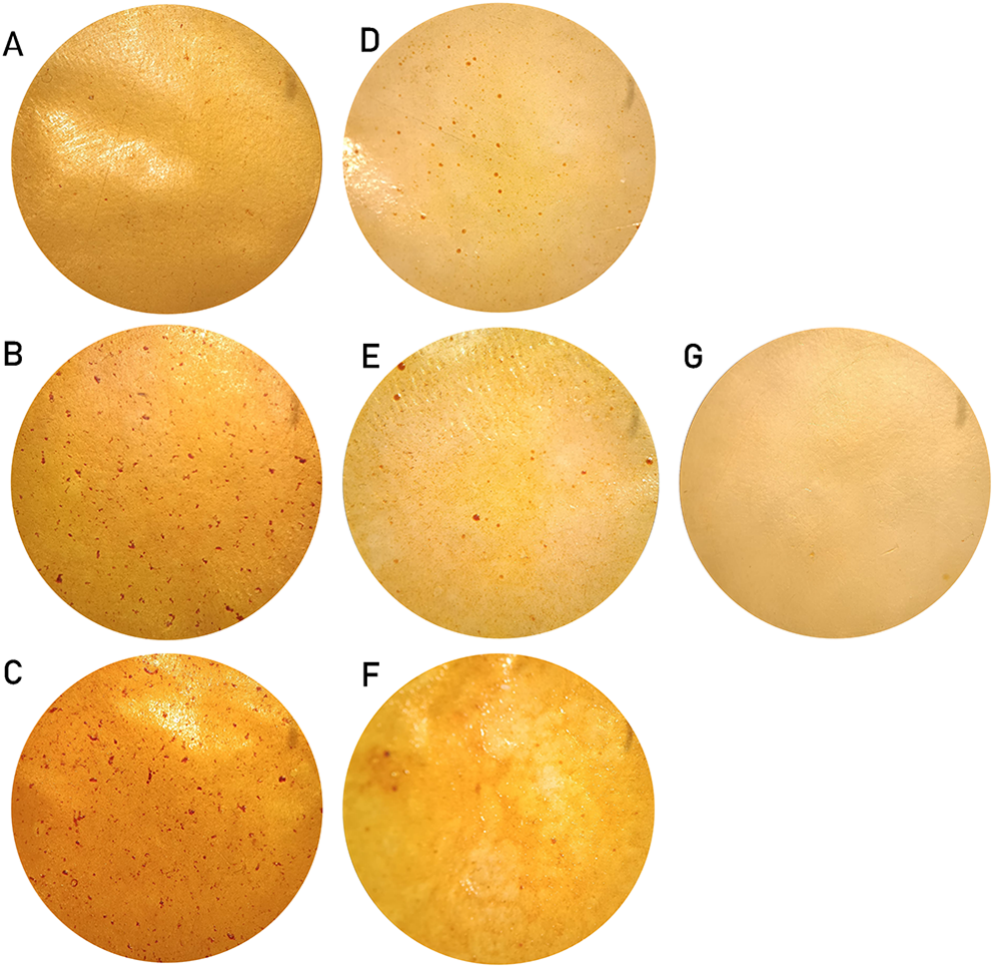
Materials produced with CMC and COMs in different concentrations (13× amplification). COM^1P^: (A), (B), and (C), for 10, 20, and 30% concentration, respectively. COM^PW^: (D), (E), and (F), for 10, 20, and 30% concentration, respectively. CMC: (G).

Visual inspection of the materials revealed the presence of darker irregular structures, more evident in the materials containing COM^1P^ especially as the COM concentration increases (Figure 2). The COMs drying process after the hydrolysis favors the formation of recalcitrant structures that are hard to solubilize before the materials casting. Therefore, the observed irregular structures on the materials surface can be constituents of the COMs that were not fully dissolved in acetone, or that precipitated or formed micelles during the mixing process. To better assess this phenomenon, we performed SEM analysis of the surface of materials containing 30% COM^1P^. The results showed embedded micellar-*like* structures (beneath the material surface) and the cross-sectional images showed pore structures (Figure S12).

The ATR-FTIR spectra of the CMC materials highlighted the presence of signature bands related to cellulose ((C–O–C) ether/ester stretch; region: 1153–1018 cm^–1^), carboxylate groups (−COO^–^; ∼1579 cm^–1^), and ester groups (stretching of C=O; ∼1736 cm^–1^) (Figure S4 and S5, Table S5, S6 and S7). The last band as well as additional bands assigned to −COO^–^ sym stretch (∼1419 cm^–1^) and – CO stretch (∼1250 cm^–1^) have been reported before^34^ for CMC dissolved in similar acidic conditions (Table S7). Finally, the band ∼3400/3300 cm^–1^ (present in all materials) is assigned to intramolecular hydrogen bonds and – OH groups.^35^ The spectra of materials comprising CMC and COMs, indicate a decrease in the carboxylate band and an increase in the ester (carbonyl) band, more evident as the COM concentration increased (Figure S4 and S5 and S11, Table S5 and S6). This result is consistent with the polycondensation reaction of OH and COOH free groups extant in both COMs and the CMC polymer chain. ATR-FTIR analysis of the materials obtained after drying (40 °C) and curing (150 °C) show the appearance of a band at ∼1180–1160 (ester C=O) indicating new ester bonds being formed (Figure S11). Additionally, the ratio between the bands at ∼1730 cm^–1^/∼2855 cm^–1^ can provide insights about the carbonyl-containing groups normalized to the CH_2_ chains. This ratio, higher at 150 °C, shows that esterification had occurred (0.88, 0.85, and 1.37 for the solution, 40 and 150 °C, respectively) (Figure S11). In addition, bands assigned to unsaturated fatty acids (e.g., olefinic = C–H stretch at ∼3010 cm^–1^) remain undetected (Figure S11) due to their low abundance, suggesting that oxidation of unsaturated lipids, if occurring, would be minor. Moreover, in the spectra of the blended materials, two bands assigned to CH_2_ asym (2920 cm^–1^) and sym (2850 cm ^–1^) stretches are noticed, which have been frequently assigned to cutin aliphatic backbone.^4,13^ The ATR-FTIR spectra of the ethanolic leachate of the materials containing CMC and 30% COM^1P^ shows features consistent with the presence of COMs and CMC constituents, with the last being more obvious (Table S5). This leachate comprised, on average, only 7.7% of the initial mass of the blended material and may, in the future, be minimized by further optimization of the polycondensation reaction conditions.

The DSC thermograms of the first heating scan of CMC (Figure S6) are consistent with an amorphous polymer (i.e., lack of a defined melting temperature),^36^ revealing an endothermal process in the 30 to 145 °C range, usually attributed to the elimination of water.^37^ The degradation temperature reported in the literature for the CMC sodium salt (∼281 °C)^38^ supports the correctness of the temperature range in the DSC analysis. In the blended materials, the thermograms show endothermic peaks similar to those of CMC for samples containing COM^1P^ (Figure S6). The materials containing COM^PW^ show a more complex endothermic behavior with multiple broad peaks. The GC-MS data of COM^PW^ showed that it is enriched in free monomers (2-fold richer than COM^1P^). In this case, the multiple endothermic peaks could be attributed not only to the water elimination but also to different crystals since fatty acids can display different crystalline polymorphs associated with different thermodynamic stabilities, as observed before in cutin-derived materials produced through polycondensation of its monomers.^39^

Analysis of the swelling of the materials showed that COM^1P^ addition reduced significantly the capacity of the materials to retain water compared to the CMC control: 1.6-fold, 2-fold, and 3-fold for blends comprising 10, 20, and 30% of COM^1P^, respectively (Table 2). The same trend was observed for the materials blended with COM^PW^ but less expressive, reaching for the materials containing 30% a 1.8-fold reduction (Table 2). These results are consistent with previous studies reporting reduced water uptake of biomaterials upon addition of cutin hydrolysates.^8,40^

**Table 2:** Absorptive Wetting/swelling Analysis and Water Contact Angle (WCA) Measurements of the Materials^a^

		swelling (%)	WCA (deg)
	CMC	70.4 ± 3.9	87.4 ± 4.1
COM^1P^	10%	44.6 ± 3.3	72.4 ± 6.4****
	20%	34.7 ± 0.3*	109.5 ± 5.5****
	30%	23.7 ± 2.5**	83.9 ± 3.5
COM^PW^	10%	52.2 ± 1.3	82.3 ± 10.8
	20%	51.1 ± 1.3	75.1 ± 8.2*
	30%	38.3 ± 2.4**	75.7 ± 5.3*

a Samples having statistical differences compared to CMC blank were marked with an asterisk (One-way ANOVA with Dunnett’s Test or Kruskal-Wallis with Dunn’s post-hoc, *p* < 0.05*; <0.01**; <0.001*** ; <0.0001****).

Finally, the surface hydrophobicity (WCA) measurements of the blended materials did not show a defined trend (Table 2), opposing that observed above for their swelling properties. The most hydrophobic surface (109.5° ± 5.5) was achieved with the blends comprising 20% COM^1P^, whereas all of the remaining blends showed similar or even lower surface hydrophobicity compared to the CMC control (87.4 ° ± 4.1). These results may reflect the heterogeneity of the materials surface (Figure 2) due to the stochastic linkage between the COMs and the CMC matrix.

### Antimicrobial Activity and Antibiofouling Properties of the Blended Materials

First, the COM^1P^ and COM^PW^ (1 mg·mL^–1^) were tested for antimicrobial activity against *S. aureus* and *E. coli* (Figure S7). At this concentration, either COM could significantly reduce the growth of *S. aureus* cells: 96.7% and 89.8% for COM^1P^ and COM^PW^, respectively. On the contrary, both COMs promoted the growth of *E. coli* cells (+166.3% and +77.6%, for COM^1P^ and COM^PW^, respectively), consistent with its ability to utilize lipids for growing and metabolize cutin monomers.^41^ Previous results also showed that cutin oligomeric mixtures were, in general, more effective against *S. aureus* than *E. coli*.^12^ Killing efficacy against *E. coli* requires esterification within the COMs.^12^ The compositional analyses (Tables S2 and S3) show that both COMs comprise monomers and small oligomeric species (mostly dimers and trimers). The *E. coli* antimicrobial results suggest that the small oligomers within the COMs are ineffective or present at a concentration below that required to kill this bacterium.

The maximum concentrations of each free monomer in COM^1P^ and COM^PW^ were compared with the published minimal inhibitory concentrations (MICs, mM) against *S. aureus* and *E. coli* (Table S8). Available MIC data for cutin-related monomers are limited, as many values derived from complex extracts or derivatives/structurally related compounds^42,43^ rather than the pure monomers themselves. Based on the reported MICs, none of the free monomers alone reached concentrations high enough in the mixtures to explain the observed antimicrobial activity against *S. aureus*, suggesting synergy between different constituents. Specifically, the aromatic compounds, which are active against both tested bacteria (Table S8), are also below the reported MICs.

The blended materials could kill *S. aureus* and *E. coli* (Figure 3). Indeed, *S. aureus* growth showed an average reduction of 72%: ranging from 65 to 75% and from 78 to 70% in the presence of COM^1P^ and COM^PW^, respectively. The blended materials with COM^1P^ were more efficient against *E. coli* (growth reduction ranging from 36 to 62%) than those with COM^PW^ (ranging from 13 to 36%). Similar results, regardless of lower efficacy, were observed with the blended materials produced using the same procedure expect that a 2-step temperature ramp (40 °C = >150 °C) was used instead of the 3-step (data not shown).

**Figure 3:**
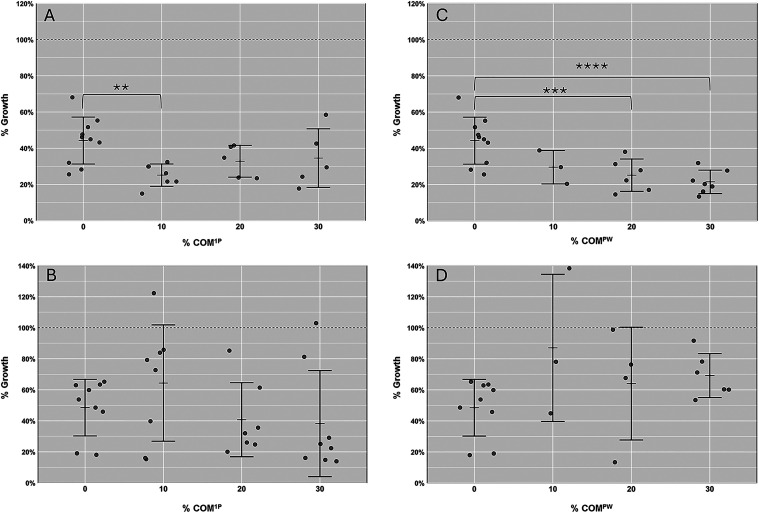
Antimicrobial activity of the materials. Bacterial growth in the presence of the materials containing CMC and COM^1P^ (A, B) or CMC and COM^PW^ (C, D) against S. aureus (A, C) and *E. coli* (B, D). The results are expressed as growth % relative to the corresponding bacterial control (without any material).

The CMC material also displayed antimicrobial activity, killing 51% of *S. aureus* cells and 67% of *E. coli* cells (Figure 3). This matrix is widely used in formulations for antimicrobial applications because of its versatility and the possibility for further modifications,^44^ and is usually accepted as a neutral polymer network.^45^ The conditions herein optimized to cast the materials (150 °C in an acidic pH) may cleave the β-1,4-glycosidic bonds in the CMC polymer chain.^46^ This could release oligosaccharides, as suggested by the chemical analysis of an ethanolic leachate of a blended material (Table S5). Oligosaccharides from CMC have been shown before to have antimicrobial potential, protecting against fruit spoilage when applied as a coating agent.^47^ Therefore, we used transwell incubation plates to test the hypothesis that the produced CMC materials can release oligosaccharides active against *S. aureus*. The CMC materials when placed into a chamber separated from the bacterial cells by a 0.4 μm membrane led to growth inhibition similar to that when in direct contact with the bacterium cells (Figure S8). As such, the observed antimicrobial activities of the blended materials (Figure 3) integrate the contribution of both COMs and CMC.

A direct comparison of the activity of the COMs, when free or blended, should not be applied. Assuming that the COMs are homogeneously dispersed in the blended materials, their maximum amount is 10-fold lower (for materials with 30%) than that tested when free (1 mg·mL^–1^); a concentration that in a previous study was found ineffective.^12^ The esterification of long-chain unsaturated fatty acids with methyl esters has been demonstrated before by Zheng et al. to hinder the antimicrobial activity against *S. aureus*.^48^ Therefore, we cannot disregard that the blending of the materials reduced, to some extent, the activity of some of the constituents of the COMs.

Unsaturated fatty acids are known to have antibiofouling properties against *S. aureus*, while saturated fatty acids have low or no effect on bacterial adhesion.^49^ In addition, polyunsaturated fatty acids like α-linolenic acid can also prevent flagellar adhesion of *E. coli*.^50^ Therefore, to complement the antimicrobial analysis, we microscopically imaged at the surface of the films the presence of adherent bacterial cells (Figure 4). The CMC control showed no adherent *S. aureus* cells, and only a few *E. coli* adherent cells. Moreover, *E. coli* could not adhere to the blended materials with COM^1P^, contrarily to *S. aureus* that formed many clusters of cells in all tested concentrations. The blended materials containing COM^PW^ showed limited adhesion of either bacterial species, regardless that higher COM^PW^ concentration potentiated adhesion, particularly of *S. aureus.* Despite *S. aureus* adherence at the material surface, adhesion is apparently lower in those containing COM^PW^ compared to those with COM^1P^. This result is consistent with COM^PW^ richness in unsaturated fatty acids (i.e., alka­(e)­noic acids) compared to COM^1P^ (Table 1). The capacity of the blended materials with COM^1P^ to block *E. coli* was systematically observed, yet it could not be directly correlated to the abundance of polyunsaturated fatty acids.

**Figure 4:**
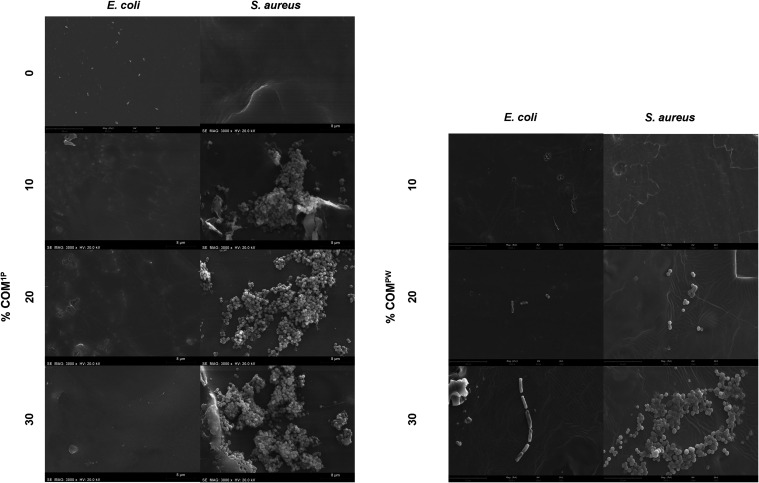
Microscopic analysis of the materials surface upon bacterial exposure. Micrographs of bacterial adhesion (*E. coli* and S. aureus) against the materials containing CMC and COM^1P^ (left panel) and materials containing CMC and COM^PW^ (right panel).

## Conclusions

Tomato pomace and tomato peels are the most used sources of the plant polyester cutin for developing biobased materials. In this study, an ionic-liquid-based process was used to isolate cutin from tomato peels, yielding a cutin polymer with all of the archetypal signatures identified before. The sustainability of the process was herein improved by recovering and reusing both the ionic liquid and the ethanol, allowing multiple isolation cycles of cutin, while lowering overall cost and enhancing process circularity.

The production of cutin hydrolysates is the starting point that enables the processability and integration of cutin polymers in applications reported across the literature. We generated COMs using two different methodologies: chemical hydrolysis (NaOH) and SCWH, aiming at the green production of oligomeric mixtures enriched with antimicrobial structures. As expected, each methodology rendered COMs with different compositional signatures, analyzed through spectroscopic and spectrometric techniques. The results showed that SCWH produced a higher percentage of monomers compared with the NaOH hydrolysis, which yielded a higher diversity of oligomers. The differences between the two COMs preparations go beyond the ratio of monomers and oligomers and reflect the relative abundance of specific monomers and oligomers. Overall, the analyses showed the complexity of both hydrolysates that comprise more than 20 chemical species.

The first question arising from this study is whether such compositional differences translate to distinct material properties. The analysis of the surface morphology and hydrophobicity, thermal behavior, and swelling showed that the materials differentiate, to some extent, in these characteristics. For example, the thermograms showed that the endothermic curves (DSC) of materials blended with COM^1P^ showed shaper peaks suggesting higher (chemical) homogeneity, which may have contributed to their more consistent antimicrobial activity. In addition, these materials displayed a clear concentration-dependent swelling behavior. Nonetheless, both materials showed surface heterogeneity, more pronounced in those blended in COM^1P^. The surface hydrophobicity did not follow the expected trend, as not all blended materials showed higher hydrophobicity compared with the CMC control. This may simply reflect their surface heterogeneity (further potentiated by the formation of micelle-like structures embedded into the films); however, one cannot disregard the potential contribution of leaching hydrophilic species from the CMC.

Regarding antimicrobial properties, both blended materials exhibited significant *S. aureus* bactericidal activity and *E. coli* antibiofouling activity, but a limited capacity to kill *E. coli*. Furthermore, only materials blended with COM^PW^ could block *S. aureus* adhesion (at some concentrations). Collectively, these results demonstrate the potential of exploring different cutin hydrolysates to tune the antimicrobial performance of the resulting materials. Neither COM alone was able to kill *E. coli*, suggesting that these hydrolysates were not sufficiently enriched in larger oligomeric species.

Integration of all data sets defines two working-hypotheses. First, the swelling of the materials (lower in all blended materials compared to the CMC-controls) is a determinant for the observed bactericidal activity against either bacterium. This is more evident for *S. aureus*, where swelling levels >40% are required for efficient killing. This suggest that swelling impacts the mobility of the free aliphatic chains, reducing their bioavailability. Second, the WCA apparently impacts *E. coli* adhesion with an ideal value >75% for efficient blockage, but not *S. aureus* adhesion. These hypotheses, which require further validation, are consistent with an incomplete esterification of the COMs to the CMC matrix, i.e., not all connective terminal groups would be esterified to CMC, hence some aliphatic chains would be bioavailable to interact with the bacterial cells.

The blended materials were prepared by a simple casting process combining COMs and CMC, primarily optimized to obtain water-insoluble materials without tailoring for a specific application. This process produced a CMC matrix that leached active oligosaccharides, which likely influenced the measured antimicrobial properties of the blended materials. Exploring alternative material-processing routes could help produce materials with minimal leaching.

The SCWH process shows potential for scaling up COMs production, which is critical to increasing the TRL of cutin-based materials. This methodology can generate hydrolysates with distinct compositional features depending on the chosen operational parameters. Thus, this provides opportunities to produce target oligomeric mixtures. Alternative green hydrolysis methods, such as other supercritical fluids, also deserve further consideration.

Our main goal was to demonstrate the potential of producing blends composed solely of CMC and cutin oligomeric mixtures and to define a robust workflow to elucidate how hydrolysate composition influences key material properties. Ultimately, this study highlights the inherent complexity of cutin hydrolysates and underscores the need for deeper chemical characterization to define structure-properties in biobased materials fabrication. Despite many remaining unknowns, our findings propose a methodological strategy for a rational design of biobased materials using renewable plant polymers. Future studies investigating polycondensation kinetics, interactions among individual constituents, and additional mechanical and physical properties (e.g., porosity, plasticity, conductivity, etc.) will be essential to tailor materials for specific applications. The use of cutin oligomeric mixtures to fine-tune material performance holds immense potential, and while this work, along with others in the literature, has only begun to uncover it, our findings already provide a solid foundation for designing fully biobased, functional materials from agro-industrial residues through green chemical routes.

## Supplementary Material



## Data Availability

All relevant data are available in the manuscript. Supplementary figures and tables are provided to support the results presented on this manuscript.

## Notes

The NMR spectra; GC–MS data; quantification tables and figures; ATR-FTIR data; SEM images and antimicrobial data (PDF)
