# Supplementary material for: Weed Out the Many Chemistries of Cutin Oligomeric Mixtures: Developing Antimicrobial Films by Blending with Carboxymethylcellulose
Source: ACS Sustain Chem Eng. 2026 Apr 15;14(16):7856–67. doi: 10.1021/acssuschemeng.6c01134 (PMC13127104; doi:10.1021/acssuschemeng.6c01134)
Supplement: Supplementary file 1 [file sc6c01134_si_001.pdf]

Supplementary Material

Weed out the many chemistries of cutin oligomeric mixtures: developing antimicrobial films by blending with carboxymethylcellulose

Rita Escórcio,[1] Artur Bento,[1] André Cairrão,[1] Enkeledo Menalla,[2] Erika Zamboni,[3] Constanza Maciel[2], Mathieu Fanuel,[4,5] Benedicte Bakan,[4] Annamaria Celli,[3] María José Cocero,[2] Cristina Silva Pereira[1],*

[1]Instituto de Tecnologia Química e Biológica António Xavier, Universidade Nova de Lisboa (ITQB NOVA), Av. da República, 2780-157, Oeiras, Portugal
[2] Department of Chemical Engineering and Environmental Technology, BioEcoUva, Research Institute on Bioeconomy, University of Valladolid, Calle Doctor Mergelina S/N, Valladolid, 47011, Spain
[3] Department of Civil, Chemical, Environmental and Materials Engineering, University of Bologna, Via Umberto Terracini, 28, 40131, Bologna, Italy
[4] INRAE, UR 1268, Biopolymers, Interactions & Assemblages (BIA), F-44316, Nantes, France
[5] PROBE research infrastructure, BIBS Facility, INRAE, F-44316, Nantes, France.

Supporting Information contains 19 pages including 12 Figures and 8 Tables.

Supplementary Tables and Figures (by order of appearance in the manuscript)

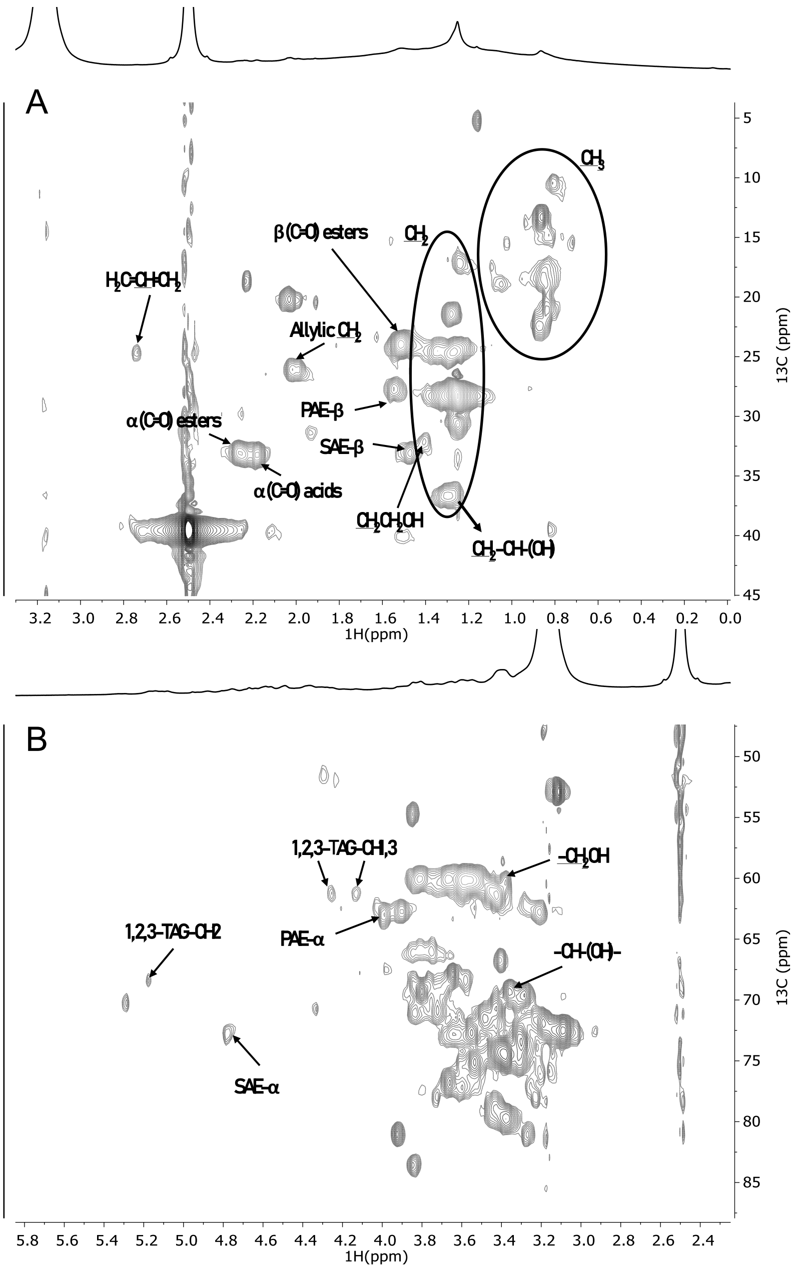

Figure S1. ^{13}C-^{1}H HSQC spectral characterization of cutin extracted from tomato peels detailing the aliphatic (A) and glycerol CH-acyl (B) regions. Some assignments (unlabeled) are uncertain or unidentified.

Table S1. Relative quantification of the ester configurations in the COMs. n.d. stands for not detected.

	SAE (%)	PAE (%)	ME (%)
COM^{1P}	52 ± 19	35 ± 14	12 ± 4
COM^{PW}	59 ± 1	41 ± 0.6	n.d.

Table S2. Quantitative analysis of the constituents of COMs by GC-MS. Results are given as mg of compound per g of starting material. The identification yields are indicated below and represent the ratio between the identified peak area and the total area of peaks in the chromatogram. Monomers that were not detected in a specific sample are labelled as n.d..

	Non-hydrolysed	Hydrolysed	Non-hydrolysed	Hydrolysed
Compound (mg/g)	**COM^{1P}**		**COM^{PW}**	
**Alka(e)noic acids**	**10.22 ± 0.18**	**16.34 ± 1.4**	**30.61 ± 17.08**	**36.71 ± 16.59**
hexadecanoic acid	3.53 ± 0.08	5.32 ± 0.9	13.73 ± 0.03	15.73 ± 0.4
9.12-octadecadienoic acid	3.37 ± 0.06	3.86 ± 0.23	12.89 ± 0.31	13.5 ± 0.07
9-octadecenoic acid	3.32 ± 0.05	3.65 ± 0.14	12.44 ± 0	13.24 ± 0.02
octadecanoic acid	n.d.	3.52 ± 0.14	12.44 ± 0	14.49 ± 0.16
**ω-Hydroxyalkanoic acids**	**78.32 ± 49.64**	**485.38 ± 62.43**	**308.81 ± 40.86**	**766.59 ± 80.12**
16-hydroxyhexadecanoic acid	n.d.	13.35 ± 4.16	28.07 ± 1.78	42.44 ± 3.17
10.16-Dihydroxyhexadecanoic acid	78.32 ± 49.64	452 ± 58.29	276.23 ± 36.05	724.15 ± 77.17
9.10-epoxy-18-hydroxyoctadecanoic acid	n.d.	20.03 ± 4.18	13.51 ± 0	n.d.
**α, ω-Alkanedioic acids**	**n.d.**	**1.66 ± 0.64**	**13.94 ± 8.82**	**13.47 ± 15.09**
Nonanedioic acid	n.d.	0.17 ± 0.12	4.95 ± 0	13.22 ± 15.77
Hexadecanedioic acid	n.d.	1.49 ± 0.52	16.19 ± 2.71	27.98 ± 0
**Aromatics**	**2.32 ± 0.98**	**21.12 ± 5.56**	**2.82 ± 0.22**	**22.58 ± 17.23**
4-hydroxybenzaldehyde	n.d.	1.47 ± 0.41	1.03 ± 0.09	17.25 ± 4.18
4-coumaric acid	1.33 ± 0.55	17.49 ± 5.69	0.54 ± 0.1	12.75 ± 6.33
Naringenin	0.99 ± 0.44	2.16 ± 1.73	1 ± 0.29	0.46 ± 0.12
**Identification yield (%)—area**	**57.41 ± 26.23**	**84.91 ± 3.91**	**52.83 ± 0.75**	**65.33 ± 1.03**

Table S3. Qualitative analysis of the oligomers detected in the COMs by MALDI-TOF.

Oligomer size	N	Oligomer_ID	theoretical_mz	Signal-to-nose ratio (S/N) COM[1P]	COM[PW]
DP2	1	hexadecanedioic acid + 16-hydroxyhexadecanoic acid	547,4544		35
	2	hexadecanedioic acid + naringenin	553,2960	166	
	3	hydroxyhexadecanedioic acid + naringenin	563,2827	175	
	4	10,16-dihydroxyhexadecanoic acid + hexadecanedioic acid	563,4494		111
	5	2x 10,16-dihydroxyhexadecanoic acid	565,4650	279	141
	6	10,16-dihydroxyhexadecanoic acid + hydroxyhexadecanedioic acid	579,4443	53	16
	7	9,10-epoxy-18-hydroxyoctadecanoic acid + hydroxyhexadecanedioic acid	605,4599	69	18
	8	10,16-dihydroxyhexadecanoic acid + hydroxyhexadecanedioic acid + 2.Me	607,4756	33	
DP3	9	2x hexadecanedioic acid + naringenin	815,4916	41	
	10	10,16-dihydroxyhexadecanoic acid + hexadecanedioic acid + naringenin	817,5073	16	
	11	10,16-dihydroxyhexadecanoic acid + hydroxyhexadecanedioic acid + naringenin	831,4866	38	
	12	2x hexadecanedioic acid + 10,16-dihydroxyhexadecanoic acid	831,6532	125	
	13	2x 10,16-dihydroxyhexadecanoic acid + hexadecanedioic acid	833,6689		18
	14	3x 10,16-dihydroxyhexadecanoic acid	835,6845		32
DP4	15	10,16-dihydroxyhexadecanoic acid + hydroxyhexadecanedioic acid + 2x naringenin	1087,5601	91	

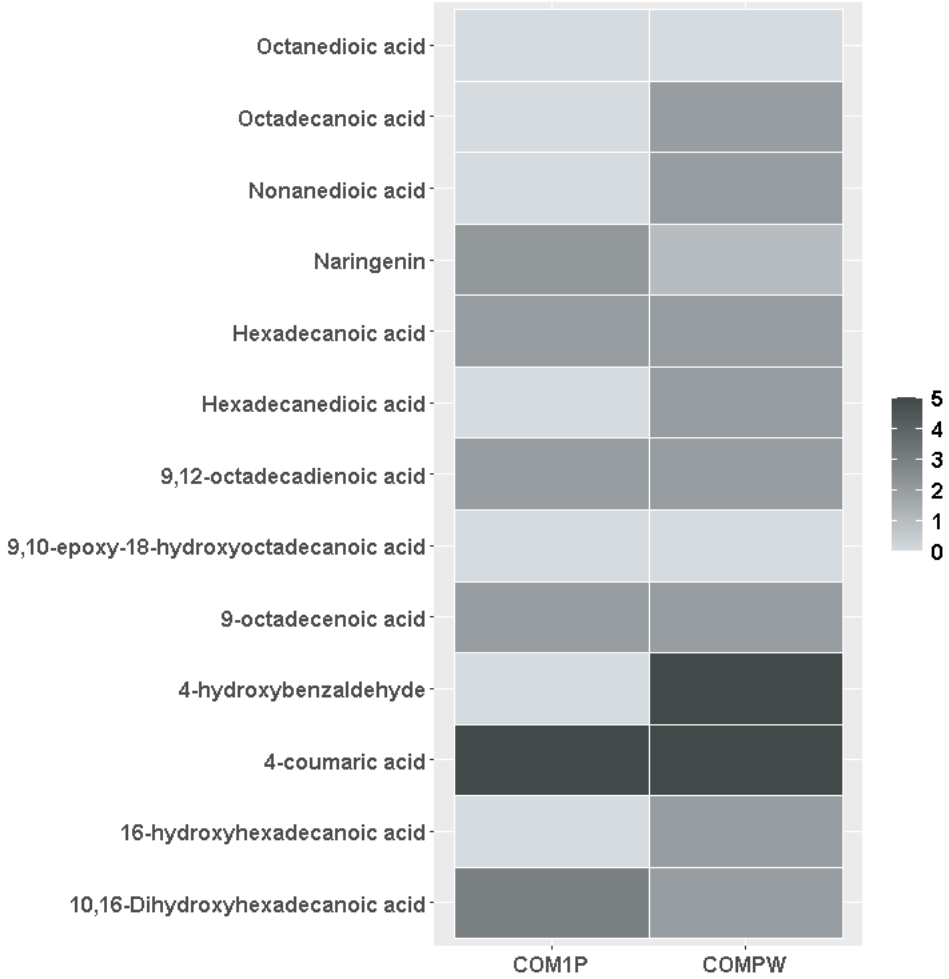

Figure S2. Heatmap of the ratios of monomers (hydrolyzed/non-hydrolyzed) present in each COM. Scaled from 0 to 5; values higher than 1 means that the monomer was linked in an oligomeric structure.

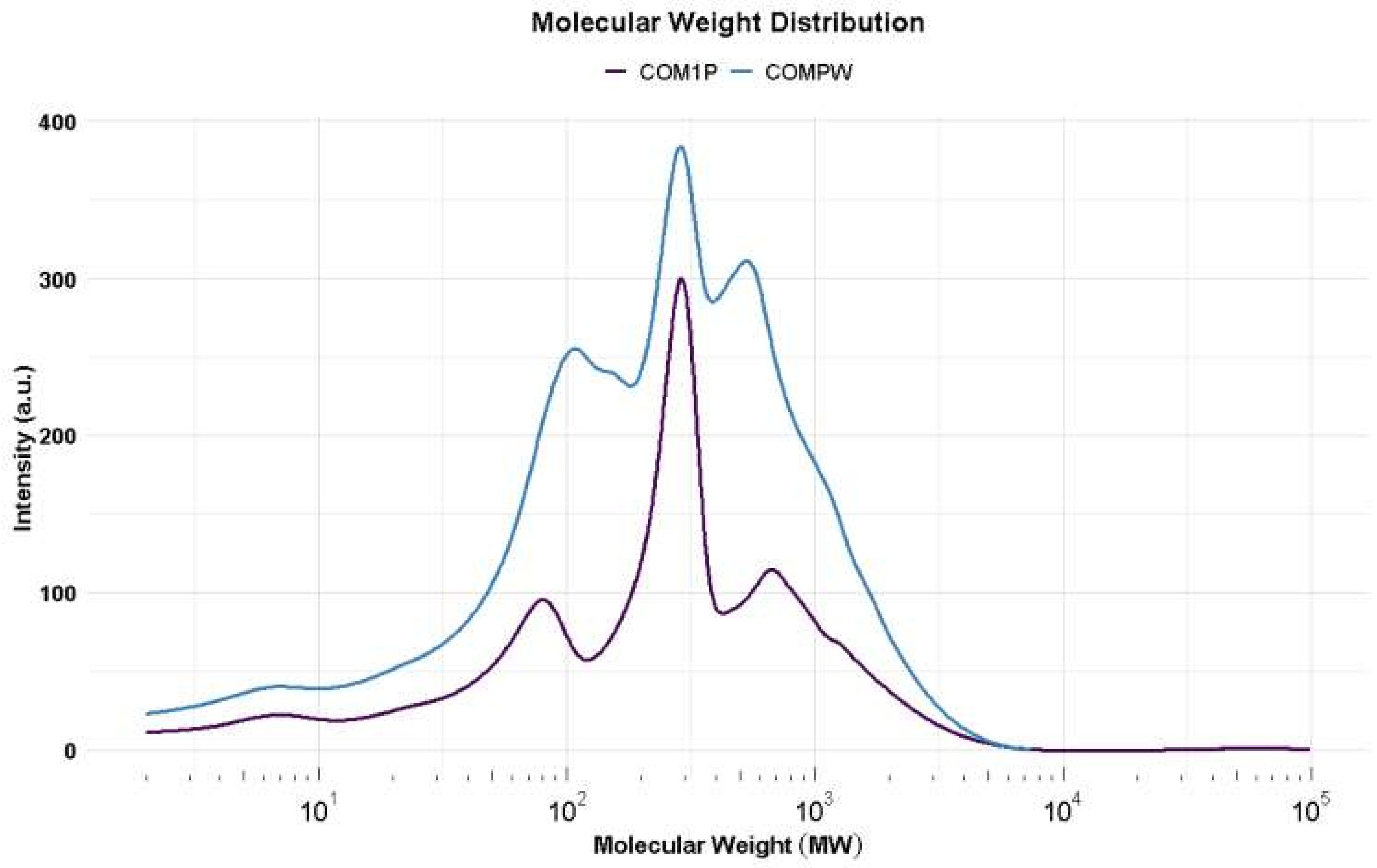

Figure S3.Molecular Weight distribution between the COM^{1P} and COM^{PW} samples.

Table S4. Quantification of ethanol washed mass and films thickness. N.d., not determined.

	COM^{1P}		COM^{PW}	
	Washed mass (mg)/film	Thickness (mm)	Washed mass (mg)/film	Thickness (mm)
CMC	~9.6 mg	0.066 ± 0.001	~9.6 mg	0.066 ± 0.001
10%	~26.1 mg	0.087 ± 0.009	n.d.	0.054 ± 0.004
20%	~15.6 mg	0.080 ± 0.011	n.d.	0.067 ± 0.011
30%	~13.7 mg	0.056 ± 0.011	n.d.	0.068 ± 0.008

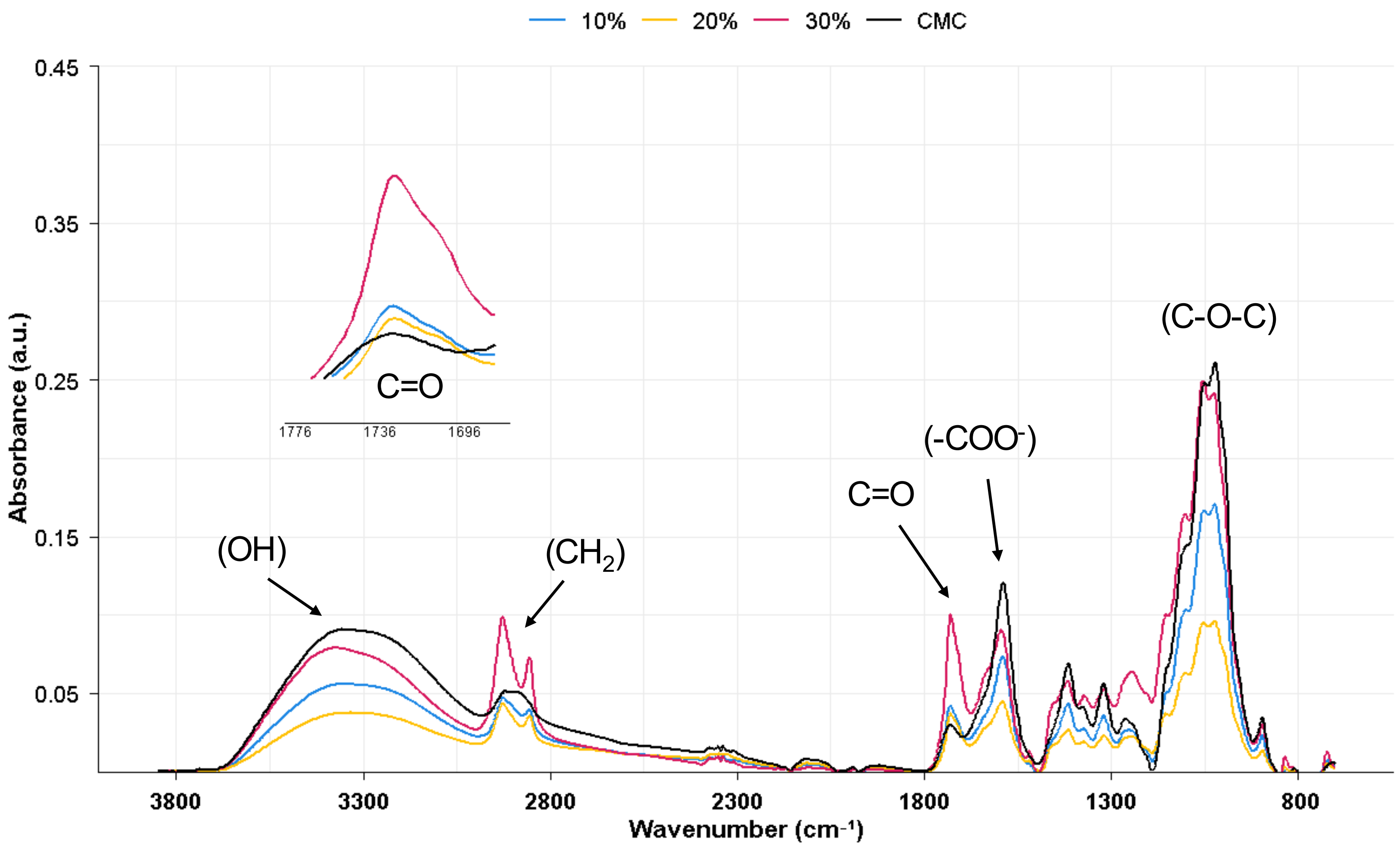

Figure S4. ATR-FTIR spectra of the materials made with COM[1P] and CMC.

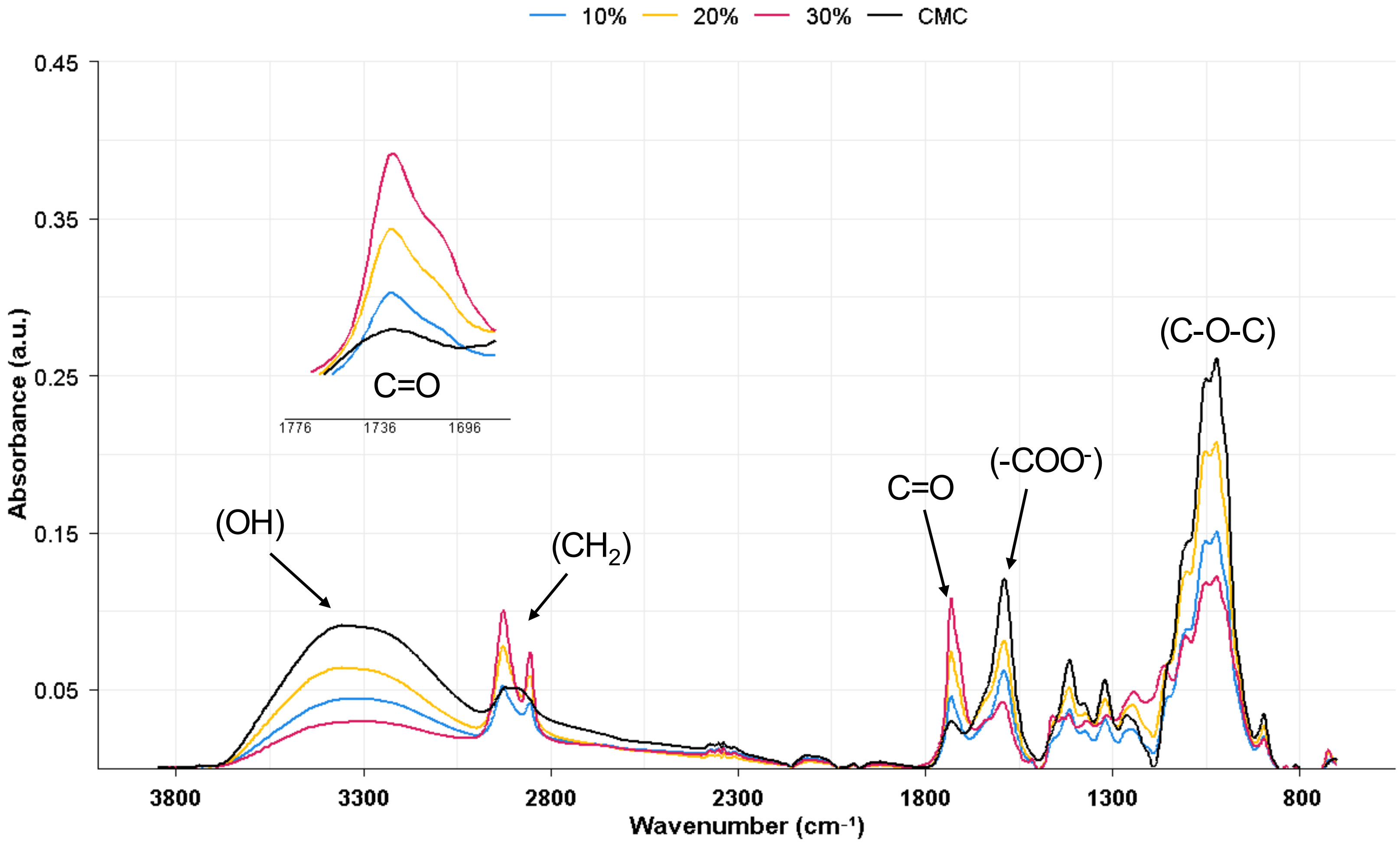

Figure S5. ATR-FTIR spectra of the materials made with COM[PW] and CMC.

Table S5. ATR-FTIR spectra of materials made with COM[1P].

10%	20%	30%	30% leachate	Assignments[1–5]
3346	3357	3379	3392	–OH stretch
2920	2929	2929	2932	CH_2 aliphatic asymmetric stretch
2850	2856	2858	2852	CH_2 aliphatic symmetric stretch
1728	1732	1726	1723	−C═O stretch
1589	1591	1591	-	$-COO^-$ asymmetric stretch
-	1519	1517	-	(C-C) aromatic (conjugated with C=C) stretch
1415	1419	1417	1417	−COO− symmetric stretch
1375	1375	1373	-	−CH and −OH coupled bend
1321	1319	1317	-	−CCH and −OCH coupled bend
1247	1245	1245	1242	−CO stretch
1153	1155	1155	1156	(C-O-C) ester stretch
1101	1099	1095	1092	(C-O-C) ether stretch or (C-O-C) ester stretch
1053	1055	1049	1054	−COC bend
1018	1024	1028	-	C(6)O bend
891	896	891	918	−CO stretching and −CH vibration

Table S6. ATR-FTIR spectra of materials made with COM^{PW}.

10%	20%	30%	Assignments[1–5]
3340	3348	3359	–OH stretch
2925	2929	2923	CH_2 aliphatic asymmetric stretch
2860	2852	2856	CH_2 aliphatic symmetric stretch
1732	1728	1728	−C=O stretch
1595	1591	1593	$-COO^-$ asymmetric stretch
-	-	1517	(C-C) aromatic (conjugated with C=C) stretch
1411	1417	1413	$-COO^-$ symmetric stretch
1369	1371	1367	−CH and −OH coupled bend
1325	1325	1317	−CCH and −OCH coupled bend
1245	1240	1245	−CO stretch
1145	1147	1163	(C-O-C) ester stretch
1097	1099	1105	(C-O-C) ether stretch or (C-O-C) ester stretch
1055	1049	1056	−COC bend
1018	1028	1018	C(6)O bend
891	891	891	−CO stretch

Table S7. ATR-FTIR spectra the CMC material.

**CMC Material**	**Assignments**[1–5]
3365	–OH stretch
2925	CH_2 aliphatic asymmetric stretch
2883	CH_2 aliphatic symmetric stretch
1724	−C═O stretch
1419	−COO− symmetric stretch
1326	−CCH and −OCH coupled bend
1250	−CO stretch
-	(C-O-C) ester stretch
-	C-O stretching
1103	(C-O-C) ether stretch or (C-O-C) ester stretch
1053	−COC bend
1026	C(6)O bend
896	−CO stretching and −CH vibration

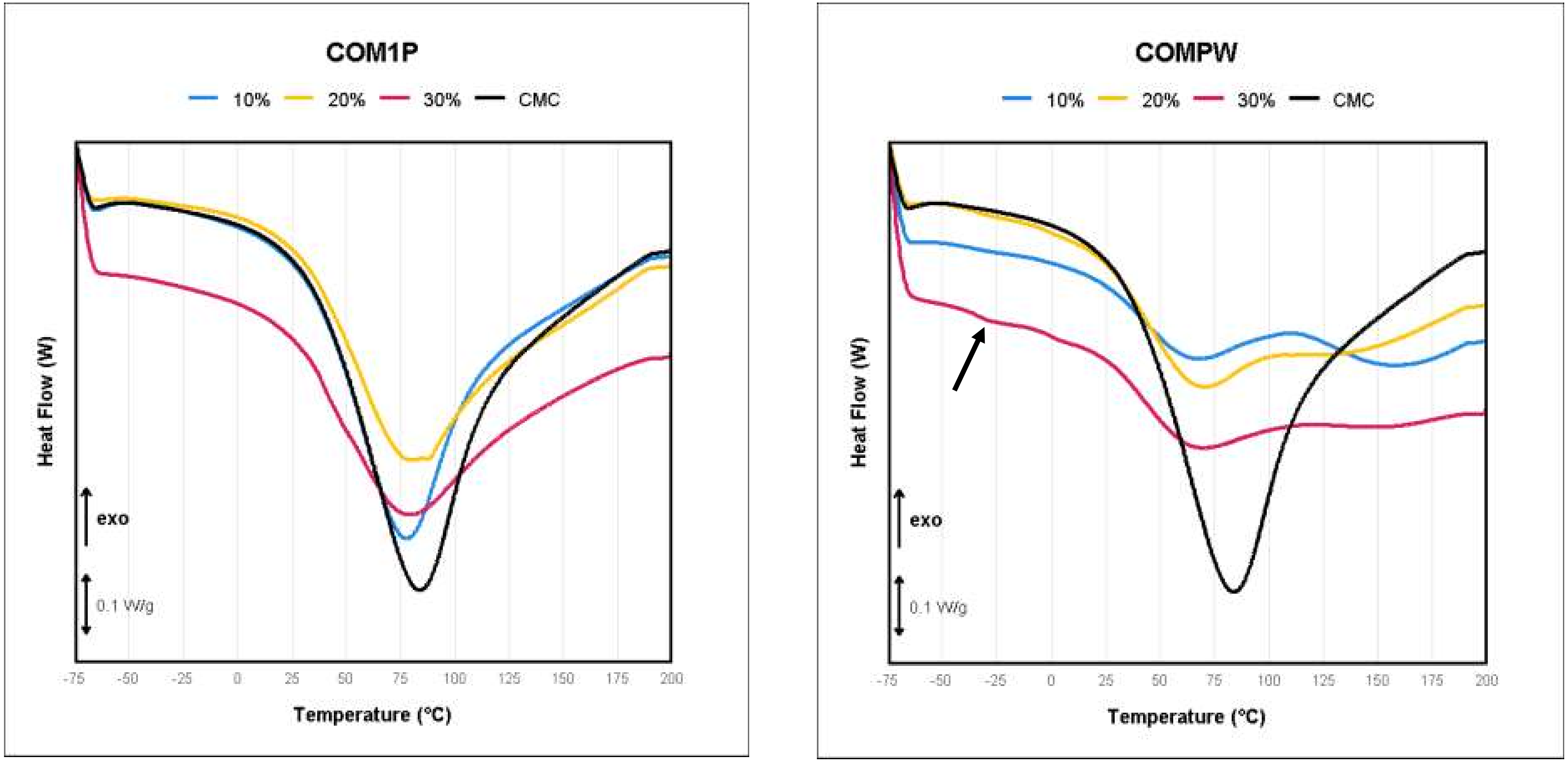

Figure S6.DSC thermograms of the materials made with COM^{1P}, COM^{PW} and CMC. Only the first heating scan is shown because the second heating scan did not show any endothermic peaks.

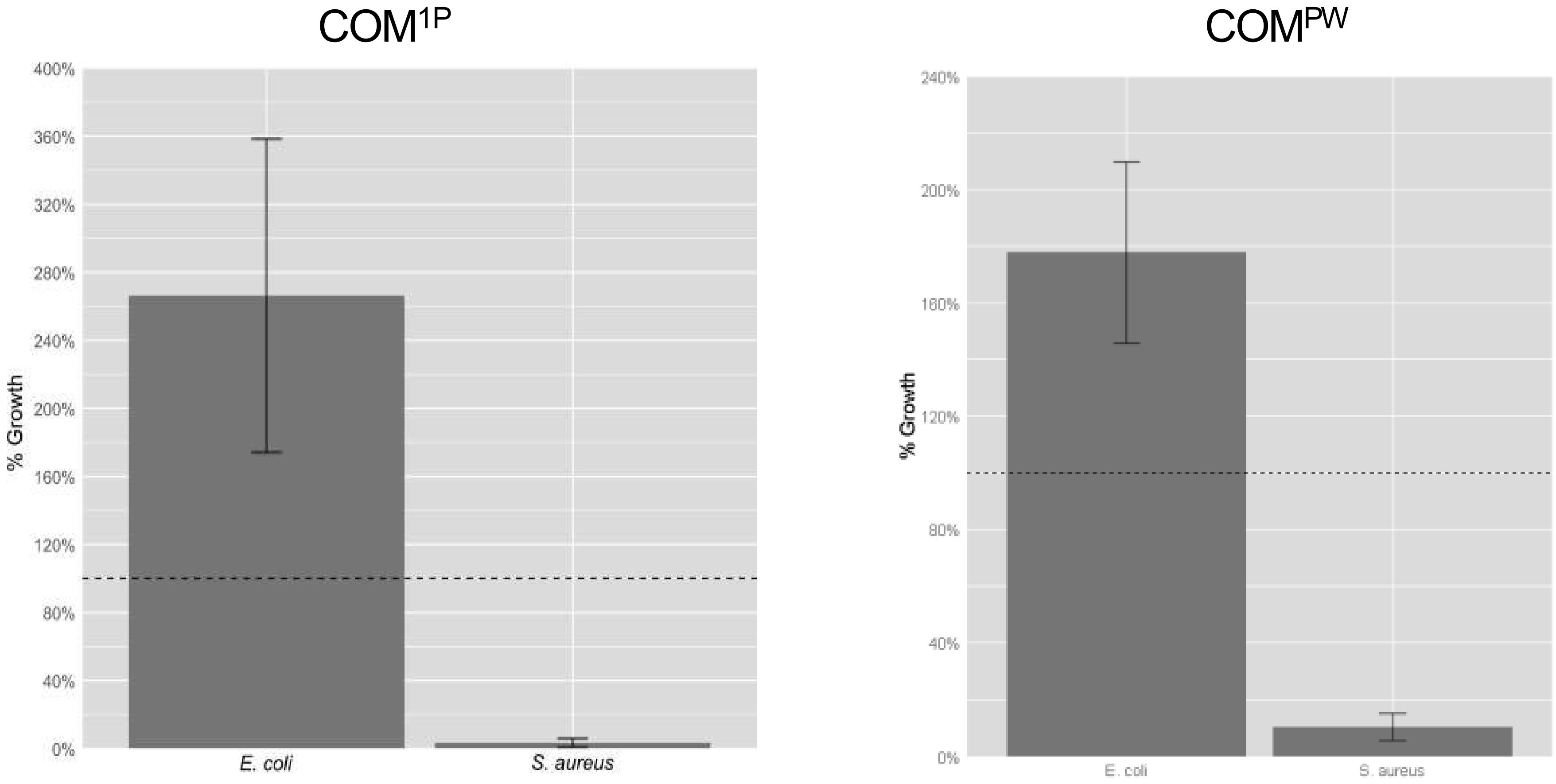

Figure S7.Antimicrobial activity of the COMs exposed directly against *E. coli* and *S. aureus.*

Table S8. Reported minimal inhibitory concentration (MIC)[6–9] of cutin monomers for *S. aureus* and *E. coli* and the concentration (mM) of free monomers in each COM sample at 1000ug/mL. as determined by GC-MS.

**Compound**	**MW (g/mol)**	**MIC (mM) *S. aureus***	**MIC (mM) *E. coli***	**COM^{1P} (mM)**	**COM^{PW} (mM)**
Hexadecanoic acid	256.43	>2 (1)	n.a.	0.014	0.054
9.12-Octadecadienoic acid	280.45	0.2 (1)	n.a.	0.012	0.046
9-Octadecenoic acid	282.46	0.4 (1)	n.a.	0.012	0.044
Octadecanoic acid	284.48	>2 (1)	n.a.	n.d.	0.044
16-Hydroxyhexadecanoic acid	272.43	n.a.	n.a.	n.d.	0.103
10.16-Dihydroxyhexadecanoic acid	288.42	n.a.	n.a.	0.271	0.958
9.10-Epoxy-18-hydroxyoctadecanoic acid	314.46	n.a.	n.a.	n.d.	0.043
Nonanedioic acid	188.19	n.a.	n.a.	n.d.	0.086
Octanedioic acid	174.2	n.a.	n.a.	n.d.	0.028
Hexadecanedioic acid	286.41	n.a.	n.a.	0.008	n.d.
4-Hydroxybenzaldehyde	122.12	n.a.	n.a.	n.d.	0.008
4-Coumaric acid	164.04	0.12 (3)	0.49 (3)	0.008	0.003
Naringenin	272.26	1.84 - ≥3.76 (2.4)	3.64 (4)	0.004	0.004

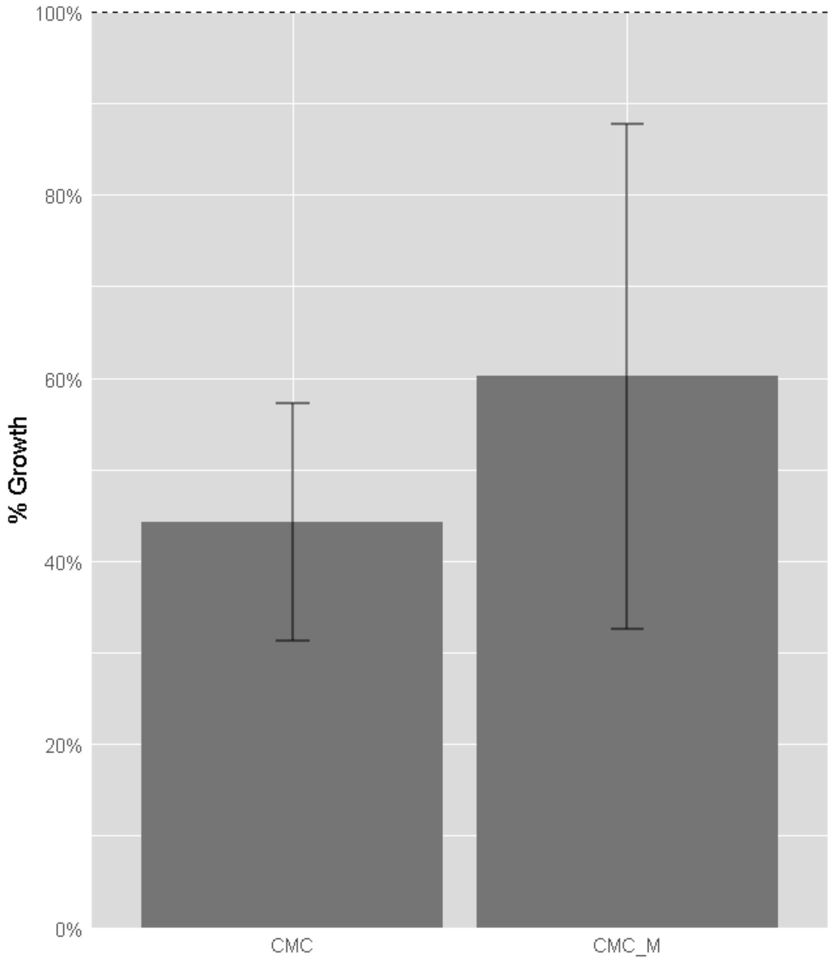

Figure S10. Antimicrobial activity of the CMC material against *S. aureus* in direct contact with the cells (left) or separated from the cells by a 0.2 µM membrane (right).

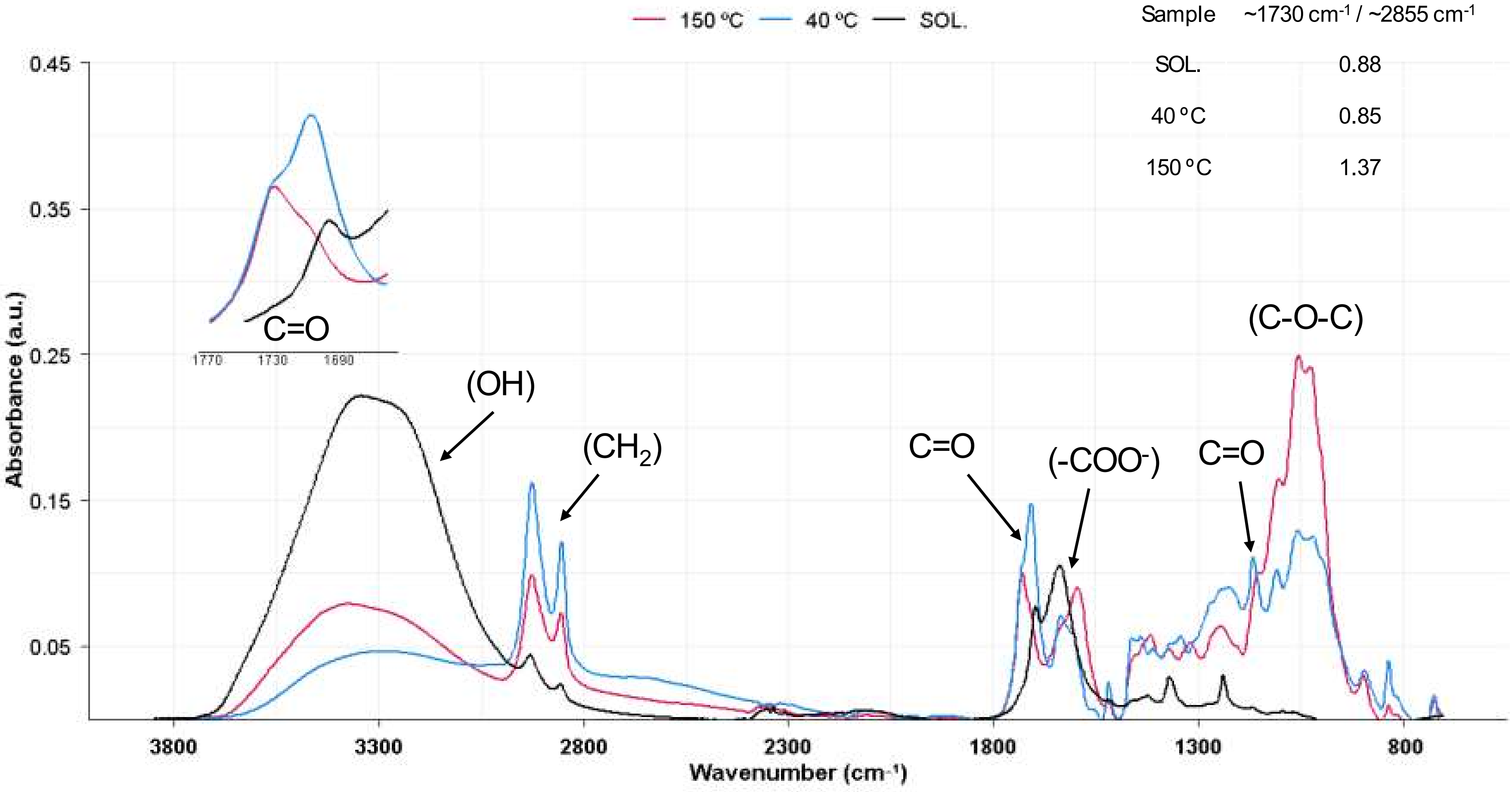

Figure S11- ATR-FTIR spectra of the materials made with 30% of COM[1P] mixed with CMC obtained along the thermal process. The black spectrum correspond to the aqueous mixture of the COMS and CMC, the blue spectrum correspond to the material after the drying process at 40 ºC and the pink spectrum correspond to the material after the curing process at 150 ºC. The table insert shows the ratio between the bands 1730 and 2855 cm^{-1}.

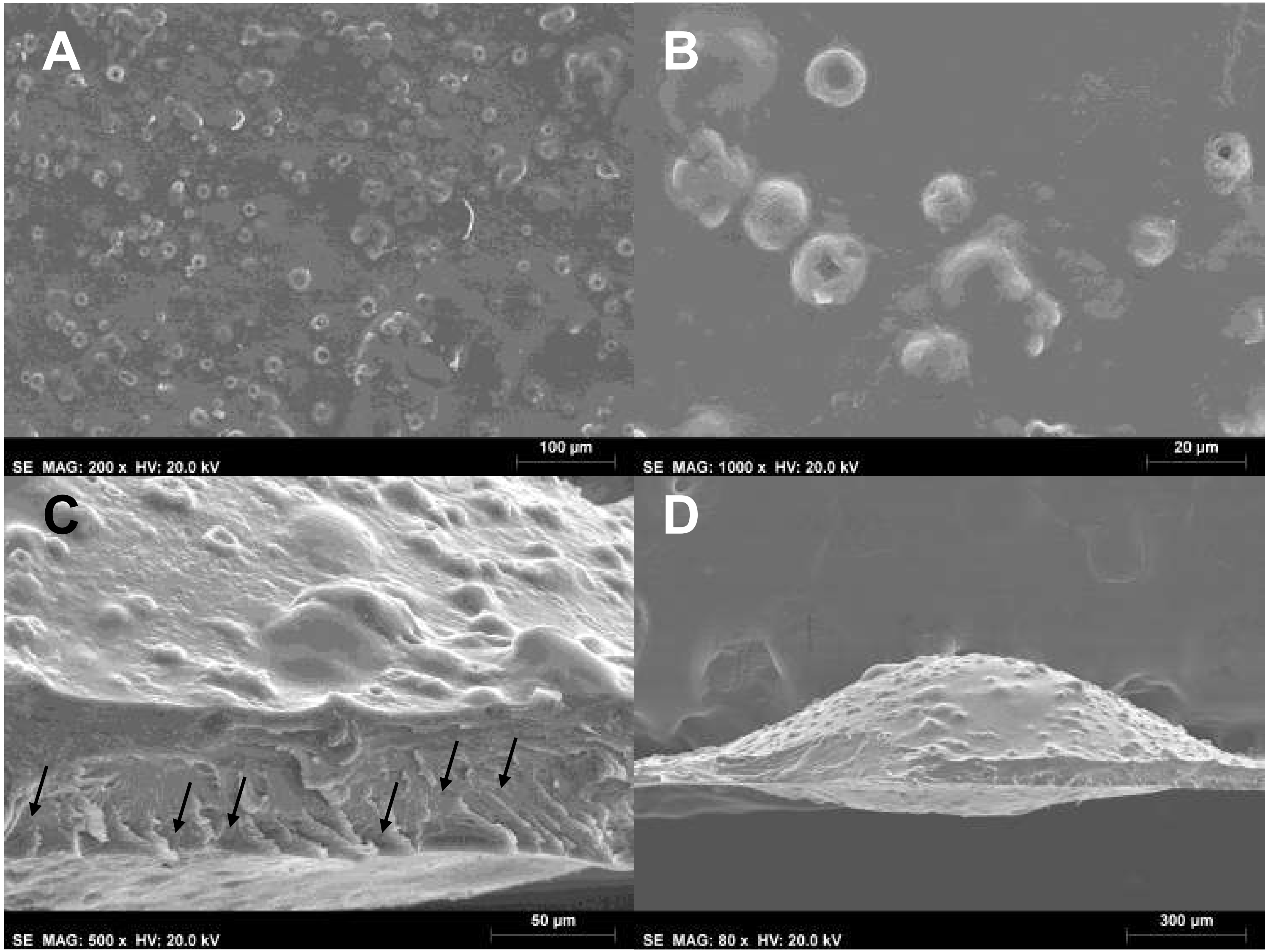

Figure S12 - SEM micrographs of 30% COM^{1P} material surface and cross sectional. A and B depict the material surface. C and D are cross sectional images of the materials, where the arrows pointing at porous structures.

Bibliography

1. Cuba-Chiem, L. T., Huynh, L., Ralston, J. & Beattie, D. A. In Situ Particle Film ATR FTIR Spectroscopy of Carboxymethyl Cellulose Adsorption on Talc: Binding Mechanism, pH Effects, and Adsorption Kinetics. *Langmuir* **24**, 8036–8044 (2008).
2. Hidayat, S., Ardiaksa, P., Riveli, N. & Rahayu, I. Synthesis and characterization of carboxymethyl cellulose (CMC) from salak-fruit seeds as anode binder for lithium-ion battery. *J. Phys.: Conf. Ser.* **1080**, 012017 (2018).
3. Aloui, H., Baraket, K., Sendon, R., Silva, A. S. & Khwaldia, K. Development and characterization of novel composite glycerol-plasticized films based on sodium caseinate and lipid fraction of tomato pomace by-product. *International Journal of Biological Macromolecules* **139**, 128–138 (2019).
4. Benítez, J. J. *et al.* Valorization of Tomato Processing by-Products: Fatty Acid Extraction and Production of Bio-Based Materials. *Materials* **11**, 2211 (2018).
5. Johar, N., Ahmad, I. & Dufresne, A. Extraction, preparation and characterization of cellulose fibres and nanocrystals from rice husk. *Industrial Crops and Products* **37**, 93–99 (2012).
6. Wang, L.-H., Zeng, X.-A., Wang, M.-S., Brennan, C. S. & Gong, D. Modification of membrane properties and fatty acids biosynthesis-related genes in *Escherichia coli* and *Staphylococcus aureus*: Implications for the antibacterial mechanism of naringenin. *Biochimica et Biophysica Acta (BBA) - Biomembranes* **1860**, 481–490 (2018).
7. Lou, Z. *et al.* *p*-Coumaric acid kills bacteria through dual damage mechanisms. *Food Control* **25**, 550–554 (2012).
8. Menezes Dantas, D. de *et al.* Naringenin as potentiator of norfloxacin efficacy through inhibition of the NorA efflux pump in *Staphylococcus aureus*. *Microbial Pathogenesis* **203**, 107504 (2025).
9. Zheng, C. J. *et al.* Fatty acid synthesis is a target for antibacterial activity of unsaturated fatty acids. *FEBS Letters* **579**, 5157–5162 (2005).
